# Biomarkers of tuber intake

**DOI:** 10.1186/s12263-019-0631-0

**Published:** 2019-04-02

**Authors:** Xiaomin Zhou, Qian Gao, Giulia Praticò, Jie Chen, Lars Ove Dragsted

**Affiliations:** 1 Department of Nutrition, Exercise and Sports, University of Copenhagen, Copenhagen, Denmark; 2 Department of Food Science, University of Copenhagen, Copenhagen, Denmark; 3 State Key Laboratory of Food Science and Technology, School of Food Science and Technology, Jiangnan University, Wuxi, China

**Keywords:** Tubers, Biomarkers of food intake, Potato, Sweet potato, Yam, Cassava, Jerusalem artichoke, Glycoalkaloids, Pyrazines, Anthocyanin, Linamarin

## Abstract

Tubers are important crops as well as staple foods in human nutrition. Among tubers, the potato in particular has been investigated for its health effects. However, except for its contribution to energy and effects related to resistant starch, the role of potatoes and other tubers in human health is still debated. In order to establish firm evidence for the health effects of dietary tubers and processed tuber products, it is essential to assess total intake accurately. The dietary assessment in most studies relies mainly on self-reporting and may give imprecise quantitative information on dietary intakes. Biomarkers of food intake (BFIs) are useful objective means to assess intake of specific foods or may be used as an additional measure to calibrate the measurement error in dietary reports. Here, intake biomarkers for common tubers, including potatoes and heated potato products, sweet potato, cassava, yam, and Jerusalem artichoke, are reviewed according to the biomarker of food intake reviews (BFIRev) standardized protocols for review and validation. Candidate BFIs for heated potato product include α-chaconine, α-solanine, and solanidine; less evidence is available to indicate peonidin 3-caffeoylsophoroside-5-glucoside and cyanidin 3-caffeoylsophoroside-5-glucoside as putative biomarkers having high potential specificity for purple sweet potato intake; linamarin may in addition be considered as a putative BFI for cassava. Other tubers also contain toxic glycosides or common contaminants as characteristic components but their putative use as intake biomarkers is not well documented. Alkyl pyrazines, acrylamide, and acrolein are formed during cooking of heated potato products while these have not yet been investigated for other tubers; these markers may not be specific only to heated potato but measurements of these compounds in blood or urine may be combined with more specific markers of the heated products, e.g., with glycoalkaloids to assess heated potato products consumption. Further studies are needed to assess the specificity, robustness, reliability, and analytical performance for the candidate tuber intake biomarkers identified in this review.

**Electronic supplementary material:**

The online version of this article (10.1186/s12263-019-0631-0) contains supplementary material, which is available to authorized users.

## Introduction

Tubers are important staple foods rich in carbohydrates and they are cultivated all over the world, save for the most arid or cold climates. The quantitatively most important tubers produced for food consumption are potato, sweet potato, yam, cassava, and Jerusalem artichoke. The potato (*Solanum tuberosum* L.) is regarded as the most important of these and is also the most important food crop in terms of the fresh product. Among starchy foods, the annual global potato production ranks fourth after rice, wheat, and maize, and the total global production was estimated at 314 million metric tonnes in 2007, while at 388 million tonnes in 2017 [1]. According to these sources, cassava ranks as the second most important tuber with a world production of 292 million tonnes, followed by yam with around 73 million tonnes of annual output [1].

The tubers are therefore important energy foods in many populations worldwide. At the same time, due to their low protein content, low quality of protein, and low levels of some micronutrients, malnutrition resulting from unbalanced intakes of tubers is also well known [2, 3], and toxic effects [4–6] have been documented as well for some tubers, while potential beneficial health effects beyond nutrition are not well documented*.* In order to utilize and document the potential nutritional and health effects of dietary tuber products, understanding of their possible preventive mechanisms and actions is important. However, the objective assessment of tuber intakes is difficult because these foods are often hidden in many dishes and preparation. Different cooking methods may also affect their health effects. The identification of tuber-specific compounds or their metabolites as potential biomarkers of food intake (BFIs) would allow objective quantification of the intake of these products in human studies.

Tubers are rarely consumed raw due to their toxicity and indigestibility. They can be processed by home cooking, at restaurants, or in the case of potatoes bought from fast-food outlets. Many potato products are prepared by the food industry, including pre-cooked potatoes, mashed potatoes, chips, French fries, etc., and potatoes are also often found as a part of ready-made meals where they are not always visible to the consumer. Some similar uses are seen for sweet potato. Moreover, potatoes and other tubers are processed in very different ways. They may be peeled or unpeeled and further heated by cooking in water, by baking, frying, deep-frying, or by advanced industrial processing to a number of frozen products or snacks as well as to flours and starches. The nutritional composition of the final tuber-derived products differs widely from essentially fat-free, low sodium, cooked products to high-fat, high-salt products rich in heat-derived degradation products. The consequent health potentials of differently processed tubers may therefore differ and for the study of health effects of different kinds of tuber products, there is a need for intake biomarkers discriminating also between the cooking and processing methods.

The objective of the present review is to provide an extensive literature review of BFIs for some common tubers and their heated or otherwise processed products according to the biomarker of food intake reviews (BFIRev) methodology [7] and to assess their current level of analytical and biological validity for use in human studies according to BFI validation criteria [8].

## Methods

For this review, we selected five of the most widely consumed tubers, namely potato, sweet potato, yam, cassava, and Jerusalem artichoke [9–12]. A systematic search was conducted in March 2017, in the following databases: PubMed [13], Scopus [14], and ISI Web of Knowledge [15]. Keywords included a combination with a group of search terms, e.g., for potato: (biomarker* OR marker* OR metabolite* OR biokinetics OR biotransformation) AND (human* OR men OR women OR patient* OR volunteer* OR participant) AND (urine OR plasma OR serum OR blood OR excretion) AND (intake OR meal OR diet OR ingestion OR consumption OR eating OR drink*) AND (Potato* OR *Solanum tuberosum* L.); all searches are presented in Table 1. The wild-card term “(*)” was used to increase the sensitivity of the search strategy. The research was limited to papers in English language, while no restriction on publication date was used in the literature search.

**Table 1:** Structured literature search terms

Operator	Database	Field	Keywords
	PubMed	All fields	biomarker* OR marker* OR metabolite* OR biokinetics OR pharmacokinetics OR biotransformation OR bioavailability OR ADME
	Scopus	Title/Abstract/Keywords	
	Web of Science	Topic	
AND	PubMed	All fields	human* OR men OR women OR patient* OR volunteer* OR participant*
	Scopus	Title/Abstract/Keywords	
	Web of Science	Topic	
AND	PubMed	All fields	urine OR plasma OR serum OR blood OR excretion
	Scopus	Title/Abstract/Keywords	
	Web of Science	Topic	
AND	PubMed	All fields	intake OR meal OR diet OR ingestion OR consumption OR eating OR drink* OR administration
	Scopus	Title/Abstract/Keywords	
	Web of Science	Topic	
AND	PubMed	All fields	For potato: Potato* OR *Solanum tuberosum* L. For sweet potato: Sweet potato OR *Ipomoea batatas* L. For yam: Yam OR Dioscorea spp. For cassava: Cassava OR *Manihot esculenta* For Jerusalem artichoke: Jerusalem artichoke OR *Helianthus tuberosus* L.
	Scopus	Title/Abstract/Keywords	
	Web of Science	Topic	

The included papers were limited to intake biomarkers of tubers and heated tuber products, and included clinical trials, randomized controlled trails, and variously designed validation studies. In regard to the exclusion criteria, all studies which focus on the effect of dietary patterns on metabolism or physiology, or effects of resistant starch and micronutrients on health, toxicology, risk assessment, or intake of other foods etc., were excluded. Search results were imported into EndNote X7 (Thomson Reuters, New York, USA) and a first screening of the papers conducted based on their titles. A second screening was then conducted based on the abstracts of the papers selected in the first screening. Finally, the papers selected from the second screening were retrieved and evaluated for their information on tuber BFIs.

In order to evaluate the specificity of the compounds found to be associated with intake of tubers and heated tuber products, an additional search was conducted. Search terms included a combination of compounds found to be associated with tuber intake and terms related to human intake and metabolism, e.g., for potato: (chaconine OR solanine OR solanidine OR alkyl pyrazines) AND (biomarker* OR marker* OR metabolite* OR biokinetics OR biotransformation OR pharmacokinetic* OR ADME OR bioavailability) AND (urine OR plasma OR serum OR blood OR excretion) AND (intake OR meal OR diet OR ingestion OR consumption OR eating OR drink* OR administration) AND (human* OR men OR women OR patient* OR volunteer* OR participant* OR subject*). The secondary search was conducted in Scifinder [16] and Google Scholar [17] besides the databases listed above. The compound database (FOODB [18] and HMDB [19]) was used as well. This second step was used to identify other foods containing the biomarkers or their precursors.

An additional unstructured search was performed to identify any additional non-nutritive compounds observed in tubers and their heated products. These compounds might form the basis for candidate BFIs, and the preliminary searches were performed for such compounds, similar to step 2 above, in order to evaluate whether they might be unique to one or more of the tubers or their processed products.

The resulting list of candidate BFIs for tubers and tuber products were validated by the recent method outlined by Dragsted and co-workers [8]. In brief, the validation assessment system has eight criteria questions, including analytical and biological validity, applied to each candidate biomarker to evaluate the usefulness of candidate BFIs for tubers within Y (yes, if the questions are fulfilled), N (no, if the questions have been investigated but they are not fulfilled), or U (unknown, if the questions have not been investigated) according to the current evidence.

## Results

**Fig. 1:**
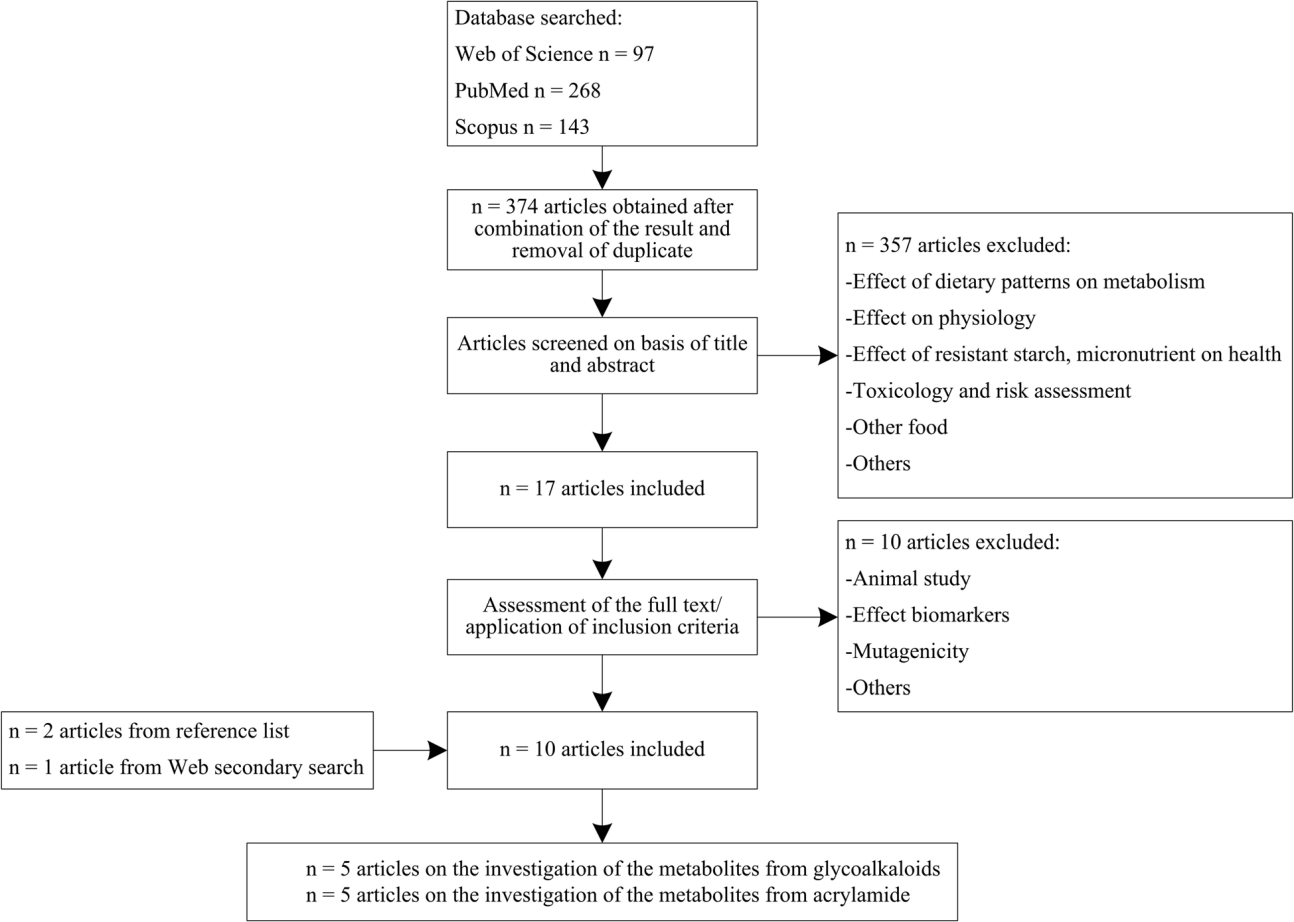
Flow diagram of structured literature search for BFIs of potato

A total of 374, 54, 64, 66, and 50 papers were retrieved from the primary database search for potato, sweet potato, yam, cassava, and Jerusalem artichoke, respectively. After the two-step screening on the basis of first the article title and then the abstract, 17, 4, 0, 21, and 0 papers were selected for full text reading for putative intake biomarkers for the five kinds of tubers. Full text reading led to removal of additional papers, leaving 7, 2, and 17 papers for potato, sweet potato, and cassava, respectively. Based on full texts of the articles, a few additional papers were identified through the reference lists from the included papers or from the secondary search, leaving ten papers dealing with BFIs of potato and heated potato product, see Fig. 1; the results of structured literature search for BFIs of other tubers have been shown in Additional file 1: Figures S1–S4. The potential specificity of the putative markers mentioned in these papers was evaluated from the secondary search and only the most promising (candidate) BFIs have been reported in Table 2 and Table 3 (markers identified in human studies for potato chips and fries intake) while other non-specific biomarkers considered during the review are listed in Additional file 2: Table S2.

**Table 2:** List of reported candidate biomarkers for tuber intakes

Dietary factor	Study design	Subjects	Analytical method	Sample type	Discriminating metabolites /Candidate biomarkers	Identifier	Primary reference(s)
Potato	Human meal study	34	Radioimmunoassay	Plasma	Solanidine	HMDB0003236	[27]
Potato products (i.e., French fries, boiled, baked or mashed potato with or without skin)	Observational study	57	Radioimmunoassay	Serum	Solanidine	HMDB0003236	[28]
	Parallel human dietary study	2					
Unpeeled potato	Parallel human dietary study	43	Radioimmunoassay	Serum (1 collection before lunch); Saliva (1 collection, eirher immediately before or after blood sampling)	Solanidine	HMDB0003236	[104]
					Total potato alkaloids (α-solanine, α-chaconine and aglycone solanidine)	HMDB0003236 HMDB0039353 HMDB0003236	
Mashed peeled potatoes	Human meal study	7	HPLC	Serum (prior and 0–25 h postdose)	Solanidine	HMDB0003236	[29]
					α-Solanine	HMDB0034202	
					α-Chaconine	HMDB0039353	
Solution with low glycoalkaloid dose; mashed potato with high glycoalkaloid dose	Human meal study	14	HPLC	Serum (prior and 0–96 h postdose)	α-Solanine	HMDB0034202	[30]
					α-Chaconine	HMDB0039353	
Sweet potato (beverage with an extract of anthocyanin)	Human meal study	6	liquid chromatography/mass spectrometry (LC/MS)	Plasma and urine (prior and 30–120 min and 24 h postdose)	Pn 3-Caf·sop-5-glc	Not in HMDB	[73]
					Cy 3-Caf·sop-5-glc	Not in HMDB	
Sweet potato (beverage with an extract of anthocyanin)	Human meal study	87	LC-ESI-MS/MS	Urine (2 h postdose)	Pn 3-Caf·sop-5-glc	Not in HMDB	[74]
Cassava	Observational study	175	Spectrophotometry	Urine	Linamarin	HMDB0033699	[105]
Cassava (Stiff porridge)	Human meal study	22	Spectrophotometry	Urine	Linamarin	HMDB0033699	[97]
Cassava (Stiff porridge made from short-soaked roots of bitter cassava varieties)	Observational study	173	Spectrophotometry	Urine	Linamarin	HMDB0033699	[98]
Cassava	Observational study	69	Thin-layer chromatography (TLC)	Urine	Linamarin	HMDB0033699	[94]
Cassava (Boiled sweet cassava)	Human meal studies	14	Spectrophotometry	Urine	Linamarin	HMDB0033699	[95]

**Table 3:** List of reported putative biomarkers for potato chips and fries intake

Dietary factor	Study design	Subjects	Analytical method	Sample type	Discriminating metabolites/candidate biomarkers	Identifier	Primary reference(s)
Potato chips (self-prepared)	Human meal study	6	LC-MS	Urine (prior and 0–72 h postdose)	AA; AAMA; GAMA	HMDB0004296; not in HMDB	[60]
Potato crisps (self-prepared and commercially available)	Human meal study	5	HPLC-ESI-MS/MS	Urine (prior and 0–24 h postdose)	AAMA; GAMA; CEMA; HPMA	Not in HMDB	[57]
Potato chips (commercially available)	Human meal study	110	UHPLC-MS/MS	Urine (prior and 0–48 h postdose)	AAMA; AAMA-sul; GAMA; iso-GAMA	Not in HMDB	[63]
Potato chips	Crossover intervention study	16	LC-MS/MS	Urine (prior and 72 h post-dose)	AA; AAMA; GAMA	HMDB0004296; Not in HMDB	[106]

AA: Unchanged acrylamide; AAMA: N-acetyl-S-(2-carbamoylethyl)-cysteine; AAMA-sul: N-acetyl-S-(2-carbamoylethyl)-l-cysteine-sulfoxide; CEMA: N-acetyl-S-(carboxyethyl)cysteine; GAMA: N-acetyl-S-(2-hydroxy-2-carbamoylethylcysteine; HPMA: N-acetyl-S-(3-hydroxypropyl)cysteine; iso-GAMA: N-acetyl-S-(1-carbamoyl-2-hydroxyethyl)-l-cysteine

**Fig. 2:**
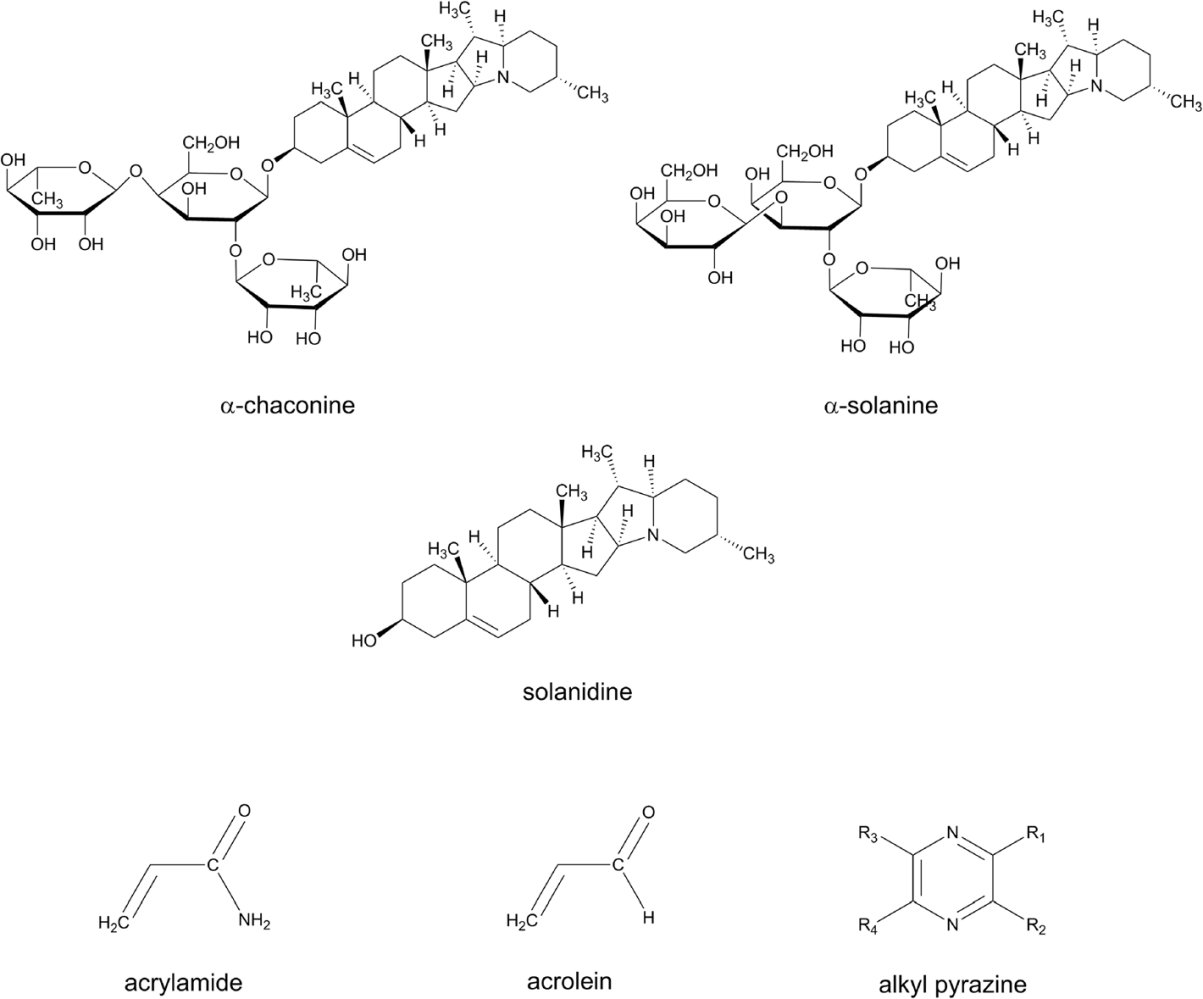
Structures of candidate biomarkers and precursors for potatoes and potato products

The secondary search for the presence of the parent compounds of these putative BFIs in other food commodities was performed along with a search for non-nutritive compounds in tubers (e.g., potatoes and potato products) that might form the basis for additional BFI candidate compounds, see Fig. 2.

### Biomarkers of food intake studies on tubers

Tubers are characterized by high contents of carbohydrates in the form of stored polysaccharides. Due to their high energy content, prevention of attack by other organisms seems important for survival and most tubers contain specific toxins to deter attack. Cultivation has often led to a reduction in contents of toxins but they are still present in modern commodities and these specialized toxins therefore form the potential basis for food intake biomarkers. A short review of the food chemistry related to our search for putative biomarkers is therefore included in the biomarker reviews for each of the tubers below.

### Potato and potato products

#### Glycoalkaloid metabolites

Solanaceae including potatoes, tomatoes (*Solanum lycopersicum* L.), and eggplants (*Solanum melongena* L.) are rich sources of glycoalkaloids. The potato glycoalkaloids, α-solanine and α-chaconine, have a trisaccharide side chain each, leading to seven hydrolysis products derived by sequent removal of the three sugar moieties from their trisaccharide side chains. The hydrolysis products, β- and γ-chaconines and -solanines and their aglycon, solanidine, may also be present in potato in small amounts; however, the intact glycoalkaloids account for 95% of the total alkaloid content [20]. The concentration is affected by genetics (e.g., variety) and environmental factors, ranging from 5.9 to 15.1 mg/100 g of potato tuber flesh [21]. More recently developed potato varieties generally have lower contents of glycoalkaloids; the joint committee for food additives of FAO and WHO (JECFA) considers amounts of glycoalkaloids between 20 and 100 mg/kg as safe [22].

It was reported that processing methods, such as boiling, baking, microwaving, and frying, affect glycoalkaloid contents. Boling reduced the α-chaconine and α-solanine levels by 6.1 and 4.1%, respectively, whereas microwaving was more destructive with a loss of 15%; the loss during deep-frying varied depending on temperature. Both compounds were stable at 150 °C, showed some loss at 170 °C, while the compounds were decreased by 35.1% for α-chaconine and 40.3% for α-solanine after 10 min at 210 °C. Significant decomposition of both compounds in potatoes starts around 170 °C [23]. Furthermore, almost all glycoalkaloid can be removed by peeling of tissue to a depth of 3–4 mm from the peel before cooking [24].

Although α-chaconine and α-solanine are the principal alkaloids present in potatoes, they have been reported in tomato plants, ranging at levels from 0.1 to 14.1 mg/100 g of fresh weight for solanine [25]; and they may also be found in alcoholic beverages according to HMDB [16]. However, others have claimed that solanine is only found in potatoes while only tomatine is found in tomatoes [26]. Thus, there is a need for further investigation to confirm the specificity of solanine and its metabolites to potatoes.

Five publications focus on potato alkaloids as human biomarkers of potato intake; three studies are human meal studies, one is a short-term intervention study, and one of the papers contains an observational and an intervention study. The biomarkers measured are total alkaloids, α-chaconine, α-solanine, or solanidine detected in human plasma, serum, or saliva. Concentrations have been determined by radioimmunoassay or HPLC after intake of peeled or unpeeled potato products.

Matthew and co-workers made a first report on detection and quantification of human plasma solanidine collected from volunteers in a hospital clinic in the UK [27]. Plasma levels determined by radioimmunoassay ranged from 0.35 to 5.00 ng/ml, and the average level was 1.56 ± 1.17 (17 males) and 1.20 ± 0.93 (27 females) ng/ml. Harvey and his group prepared different kinds of potato products, including jacket potato, roast, boiled, and mashed potatoes, or French fries, with or without skin, and recorded the potato consumption of subjects daily for 1 month, using arbitrary units assigned to each product to reflect its corresponding level of glycoalkaloids [28]. The serum samples, collected before lunch, were analyzed for solanidine by radioimmunoassay. There was a significant correlation between serum solanidine concentration and alkaloid intake from potatoes. The average level of solanidine in males was 10.8 ± 5.4 ng/ml, whereas in females it was 7.9 ± 4.3 ng/ml. Serum levels declined to 0.5 ng/ml after potato was avoided for 2 to 3 weeks. In addition, solanidine may have an ability to bind to blood constituents as the free sterol, thereby delaying excretion.

Another study reported that the mean serum solanidine level is higher for the Swedish consuming potatoes with a higher level of glycoalkaloids than subjects eating their normal diets, and only glycoalkaloids and solanidine were present in serum after potato consumption, while no intermediate β- or γ-glycoalkaloids were found [29].

Hellenäs and co-workers were also the first to apply HPLC to study the kinetics of potato glycoalkaloids in humans [29]. Using a cyanopropyl column for initial fractionation, glycoalkaloids and solanidine from the relevant fractions were subsequently separated on a silica column and quantified. For toxicokinetics, they collected blood serum samples from seven subjects 1–25 h after a single meal with peeled mashed potatoes. The concentrations of α-solanine and α-chaconine increased after potato consumption; levels peaked at 4–8 h, and the biological half-lives for these two compounds were 10.7 and 19.1 h, respectively. The blood serum concentration was from 6 to 21 ng/ml for α-chaconine and 3 to 11 ng/ml for α-solanine, while solanidine showed a lower level < 4.0 ng/ml. Mensinga et al. [30] designed a human study where 14 subjects received 1 of 6 different dose levels; the doses administered were 0.30, 0.50, 0.70 and 0.95, 1.10, 1.25 mg of total glycoalkaloids [kg body weight (BW)]^−1^ provided by solutions with glycoalkaloids and mashed potato, respectively. Increased doses led to higher peak serum concentration levels (*C*_max_) of both α-chaconine and α-solanine and the relation between *C*_max_ value and absolute dose was positive.

No recent papers were found reporting the detection of potato glycoalkaloids in blood collected from healthy subjects exposed to heated potato products, and it seems that α-chaconine and/or α-solanine and/or solanidine may be considered as candidate biomarkers for potato intake; however, there is not enough evidence from human studies to assess sensitivity and specificity.

#### Other potato compounds

Several potato-derived metabolites from Additional file 3: Table S1 were not considered suitable as precursors of BFIs due to their apparent lack of specificity to potato. Phytochemicals such as phenolic acids and certain flavonoids are important components of potatoes as recently reviewed by others [31, 32]. However, none of these seem specific to potato and contents are also highly variable depending on variety and breeding conditions.

Chlorogenic acids are reported as the main phenolic compounds in potatoes, comprising more than 90% of phenolics [33, 34]. Their metabolism in humans is complex, just like metabolism of most other phenolics, which also exist in many types of fruit [35]. The known potato phenolics were therefore not considered sufficiently specific to be evaluated as candidate potato intake biomarkers and were omitted in Table 2.

Among the flavonoids, anthocyanins are present at high levels in tubers of colored cultivars, showing a much lower concentration in the yellow and white cultivars [36]. Anthocyanins in potatoes are glucosides primarily originating from six anthocyanidins—pelargonidin, petunidin, peonidin, malvidin, delphidin, and cyaniding [37]. Red-fleshed potatoes contain acylated glucosides of pelargonidin [38, 39], while acylated glucosides of pelargonidin, malvidin, penunidin, peonidin, and delphindin are additionally present in purple-fleshed potatoes [37, 40]. According to Fossen and Andersen, two novel anthocyanins of purple-fleshed (cv Congo) potatoes were confirmed, which consisted of ferulyl gluco- and rhamno-pyranosides of malvidin and petunidin [41]. However, none of these anthocyanins seem specific to potato.

Also many carotenoids, ascorbate, and minerals are too common in other foods to be considered specific markers of potato intake. Only three carotenoids seem common in potato, lutein, zeaxanthin, and violaxanthin, while the carotenoids, neoxanthin and antheraxanthin, have been reported in some studies as well [42]. Intake of these carotenoids may also come from other food sources [43] and finding these carotenoids in blood would therefore not necessarily be related to potato intake. No studies were found specifically addressing the carotenoid profile in blood following long-term high level intakes of potato, so besides the lack of evidence for specificity based on food science, there is also a paucity of studies on these carotenoids in humans following potato intake.

#### Markers of processed potato intake

Potatoes are heated before consumption and depending on the heating method aroma compounds may be formed, including pyrazines, oxazoles, thiophenes, etc. of which pyrazines are particularly abundant in heated potato products [44]. These compounds are mainly formed by carbohydrate, amino acid, and lipid degradation by Maillard reactions and their formation process may be affected by minerals and vitamins [45].

In boiled potatoes, the number of flavor compounds identified in one study ranged from 140 to 182, depending on factors such as cooking conditions and preparation methods, e.g., whether the potatoes were peeled or unpeeled. The major volatile compounds of boiled potato flesh include methional, aliphatic alcohols and aldehydes, thiols, disulfides, and methoxypyrazines [46, 47]. Additional flavor compounds have been identified in baked potatoes, mainly formed by lipid degradation, Maillard reactions, and sulfur amino acid degradation [47]. In general, the most important flavor compounds in baked potatoes are pyrazines [48] and methional [48–50], but the former is present at quantitatively very low levels. In fried potatoes, van Loon et al. have identified most of the 122 compounds, they observed as produced from sugar or lipid degradation and/or Maillard reactions [51]. Pyrazines, especially 3-ethyl-2,5-dimethylpyrazine, fatty medium-chain length dialdehydes along with methylthiol, are reported as dominant and abundant flavor compounds in potato fries and chips [48].

Consequently, the most characteristic flavor compounds formed in heated potatoes are short-chain aliphatic aldehydes, Strecker aldehydes of medium-chain length, and various alkyl pyrazines. The aldehydes are observed after any heating while the pyrazines are most characteristic of potato fries and chips. Pyrazines could therefore potentially form the basis for biomarkers related to heating.

Acrylamide (AA) is another Maillard reaction product formed when potatoes are baked, fried, or deep-fried. The compound is not specific to potato but also present in other baked or roasted products of plant origin such as coffee or bread [52, 53]. However, French fries and potato chips are abundant sources, although industrial variety selection and processing has reduced the contents in these foods considerably over the last 15 years [53]. AA is metabolized to the epoxide, glycidamide (GA), and both compounds are electrophiles forming adducts with macromolecules [54] and glutathione [55]. They may therefore be measured as mercapturates in urine or as macromolecular adducts accumulating over time periods that depend on the half-life of the macromolecular structures. Especially hemoglobin adducts (AA-Hb and GA-Hb) have been measured after AA exposures [56]. Hemoglobin adducts are not specific to potato since they can result also from environmental or occupational AA exposure, smoking, as well as other dietary AA exposures, including coffee intake. Mercapturic acid metabolites of AA in urine represent short-term exposure, whereas AA-Hb from blood represents average exposures over several months. GA-Hb can indicate the genotoxic GA dose and is affected by the individual susceptibility to AA activation. Hemoglobin adducts may therefore be less suitable as a dietary exposure marker.

Acrolein (AC) is another electrophile known to be present in potato chips and fries due to formation in the cooking oils during heating [57]. This compound also forms glutathione conjugates and is extensively excreted in urine as mercapturates, and AC is not specific to potato since it is present in heated foods in general and also comes from environmental and endogenous formation [58] . However, the environmental AC emissions, e.g., by combustion of petroleum fuels and biodiesel, do not have significant difference [58]. Taken together, the urine metabolites of AC could be considered as one of combined BIFs of potato intake if the subjects are healthy non-smoker and the emissions of environmental AC are controlled.

Exposure to AA, AC, and their mercapturic acid metabolites has been investigated extensively in recent decades. Several studies have identified mercapturic acid metabolites of AA and AC as markers of potato products intake. These include *N*-acetyl-*S*-(2-carbamoylethyl)-l-cysteine (AAMA) and *N*-acetyl-*S*-(2-carbamoyl-2-hydroxyethyl)-l-cysteine (GAMA) which are considered as prominent biomarkers in urine of AA intake and GA formation, respectively [59]. These two compounds are reported to be excreted in urine accounting for 50–60% of the administrated dose in animals [60–62]. Wang and his group in a rat study also observed *N*-acetyl-*S*-(1-carbamoyl-2-hydroxyethyl)-l-cysteine (iso-GAMA) as an AA excretion product [63]. All mecapturates reached their peak level within 3 h, and the excretion level of AAMA was higher than the others during the initial formation stage; AAMA then decreased relatively fast while the levels of GAMA and iso-GAMA only slowly declined during the elimination stage, indicating longer elimination half-lives of the glycidamide mercapturates. The toxicokinetics of AA in urine during a human potato chip meal study has also been investigated. The average levels of AAMA, GAMA, iso-GAMA, and *N*-acetyl-*S*-(2-carbamoylethyl)-l-cysteine-sulfoxide (AAMA-sul) decreased in the initial 2 h and then exponentially rose in the next 12 h, followed by apparent first-order decline. These studies indicate that AAMA is an early and principal biomarker among the four urinary mercapturates with a half-life of 14.6 h.

*N*-acetyl-*S*-(3-hydroxypropyl) cysteine (3-HPMA) and *N*-acetyl-*S*-(carboxyethyl) cysteine (CEMA) are two metabolites from AC in urine. Five male Caucasian non-smokers were exposed to acrolein by consumption of commercially available potato crisps with a content of 26.5 ± 2.4 μg/kg (mean ± standard deviation). The results showed that urinary 3-HPMA levels were increased exponentially in the initial 4 h and then slightly declined at 8 h after test meal intake. The total urinary 3-HPMA and CEMA levels were much higher than AAMA and GAMA. This is remarkable since isotope dilution headspace GC/MS determined that the AA content of potato crisps far exceeded the content for AC by up to ten times. The results indicate that potato crisps may contain higher content of AC than that of AA, but it is not well known whether this may also apply to other heated foods [63]. Thus, it is important that more reliable analytical studies are done to resolve this.

Due to their abundance, the alkyl pyrazines, possibly in combination with markers of AA, GA, or AC, may therefore be the most promising compounds to form the basis of markers specific to fried and deep-fried potato product intakes. As specific biomarkers for potato or heated potato products, they may be useful in combination with other markers (e.g., glycoalkaloids) to indicate the heating methods (baking, frying, or deep-frying methods), see Table 3.

Potato cultivation and processing practices may also be the basis for biomarkers, e.g., from potato-specific treatment agents. Khakimov et al. [64] reported that 2,6-diisopropylnaphtalene (2,6-DIPN), an anti-sprouting agent for stored potatoes, discriminated between subjects consuming an average Danish diet and those on a so-called New Nordic diet due to the different intakes of conventional potato-based processed products leading to a higher excretion level of 2,6-DIPN in subjects on the conventional diet. Though 2,6-DIPN is not a compound from potato products and their metabolites, it is almost only used as an anti-sprouting agent for potatoes destined for industrial processing and may therefore be considered as a marker indicating intake of processed conventional potato products.

#### Sweet potato

Sweet potato (*Ipomoea batatas* L.) belongs to the Convolvulaceae family (morning glory); its roots are rich in carbohydrates (ca 80%), primarily in the form of starch (ca 50%), which is extracted and valued as a highly important ingredient in food industry due to its special physicochemical properties [65]. Sugars such as sucrose, maltose, and glucose, are responsible for the sweet taste of the root. Pectins, hemicelluloses, and cellulose represent other polysaccharides with a lower content in sweet potato roots. The total protein accounts for approximately 5% of the dry matter in sweet potato [10].

The yellow and orange colors of the tuber skin and flesh are due to carotenoid pigments. Orange-fleshed sweet potato has a high level of total carotenoids and in particular β-carotene, and sweet potato is a considered a good source of pro-vitamin A to reduce vitamin A deficiency [10, 42, 66]. In addition to carotenoid pigments, acylated anthocyanins are responsible for the red, purple, or blue flesh colors in some varieties of sweet potato, and acylated derivatives of cyanidin and peonidin are the predominant glucosides [10].

As already mentioned, phytochemicals such as common anthocyanins and carotenoids are widespread in plant foods and thus is not specific to sweet potato. 4-Ipomeanol has been reported to be present only in damaged sweet potato tubers infected by the mold *Fusarium solani* [67] and may therefore also be of more limited use as a biomarker.

Only two papers were found on biomarkers for sweet potato and both the focus was on acylated anthocyanins in urine and plasma after purple sweet potato (PSP) intake. PSP contains a high level of anthocyanins, and Ayamurasaki is one of the Japanese selected tuber varieties, which has the largest contents of anthocyanins [68]. In order to elucidate the contribution of PSP intake to physiological functions, such as provitamin A activity, radical scavenging [69, 70], and antimutagenicity [71, 72], Harada and coworkers investigated the PSP anthocyanin bioavailability [73]. Six healthy volunteers drank a beverage prepared from PSP while collecting blood and urine samples for determination of two major anthocyanin components, peonidin 3-caffeoylsophoroside-5-glucoside (Pn 3-Caf·sop-5-glc) and cyanidin 3-caffeoylsophoroside-5-glucoside (Cy 3-Caf·sop-5-glc) by LC/MS. It was reported that these anthocyanins reached the highest level in plasma 90 min after consumption, showing that the PSP anthocyanins were directly absorbed into the blood stream at an early stage. The recovery rate in the urine was from 0.01 to 0.03% in 24 h.

Pn 3-Caf·sop-5-glc was also identified by another intervention study in which 87 healthy volunteers were recruited to consume a beverage with 1 of 3 different levels of PSP anthocyanins [74]. However, the content of acylated anthocyanin in urine did not show any dose-response relationship, which might be caused by interactions with the carbohydrate content and carbohydrate composition of the beverage or possibly indicate saturation kinetics even at low intake levels.

Acylated anthocyanins are widely distributed in plants, including grapes, berries, red cabbage, etc.; however, Terahara N et al. [75] have identified a specific structure of acylated anthocyanins from the root of PSP, *Ipomoea batatas* cv Yamagawamurasaki, which is cultivated in Japan, namely 3-O-β-(6-O-(E)-Caffeylsophoroside)-5-O-β-glucopyranoside, a basic structure of acylated anthocyanins for PSP, which has not been identified in other major foods. Moreover, there is a paucity of information about human metabolism of acylated anthocyanins and further research is needed to investigate this marker as a putative BFIs for PSP intake.

#### Yam

Cultivated yams, the tubers of certain *Dioscorea spp.*, play an important role as staple food for millions of people in many temperate, tropical, and subtropical world regions [10]. Even more interestingly, yam tuber has been utilized not only as a reliable food in times of famine or scarcity but also as a traditional medicine since it contains some pharmacologically active compounds [76]. The major carbohydrate of yam tubers is starch, which can account for up to 85% on the basis of dry weight [77] and is important for the nutritional quality of food products made from yam tubers [78].

Tubers or rhizomes of some yam species are known to produce steroidal C_27_ saponins [76, 79–81]. The extracted diosgenin, the aglycone of the saponin, dioscin, has been exploited as a material for the commercial synthesis of pregnenolone and other steroid products with the aim of producing combined oral contraceptives [82]. Diosgenin precursors may exist at levels up to 20 mg/g in some *Dioscorea* species [83]; however, reported analyses of yam for food production is scarce with levels more than 100 times lower [84]. Diosgenin may also be found in carrot, wild carrot, *Allium* species, and fenugreek according to FOODB [18] and HMDB [19], and it is therefore not specific to yam tubers. The levels in carrot are reported at 5.7 mg/g which is lower than that in some yam species [18]; several diosgenin glycosides have also been reported in wild garlic; however, this plant may not be commonly consumed. The presence in other *Allium* species has not been reported and needs investigation. Fenugreek seed has been reported to contain levels of 4–8 mg/g [85] and is mainly used as a spice and therefore consumed in much lower amounts than yam. Due to the variable and potential null level of diosgenin in yam and the potential contribution, albeit limited, from carrot, fenugreek, and possibly *Allium*, the potential of diosgenin as a BFI for yam intake is questionable and its robustness needs substantiation in human studies. No studies were found investigating biomarkers of yam intake.

#### Cassava

Cassava (*Manihot esculenta*) is an important potato-like food and a drought-tolerant staple grown in tropical and subtropical areas. Cassava is to many populations in Africa as the rice to Asian people, or potato and wheat to the European countries. The roots of cassava contain predominantly carbohydrates, representing 80 to 90% of dry matters, and they have higher contents than potato [3]. The major part of the carbohydrate is starch, accounting for 80% and the main remaining forms are sugars. Toxic cyanogenic glycosides are present in cassava; these compounds can liberate cyanide, which has acute toxic effects [86]. Longer-term exposures to lower levels of cyanide and its primary metabolite, thiocyanate, have additionally been associated with a range of negative health outcomes, including goiters and paralysis [87, 88].

Cyanide can be released from cassava by two related cyanogenic glucosides, linamarin accounting for 95% and lotaustralin comprising 5% [89, 90]. Free linamarin may be directly absorbed and excreted into the urine in humans [91]. However, if it is bound in the food matrix, it is likely to be degraded to cyanide by gut microbes.

Grating and crushing are very important processes in reducing cyanide levels because damage to the cassava tissue allows direct contact of glycosides with the enzyme, linamarase, an endogenous enzyme present in the cassava cell walls producing HCN. After liberation, the cyanide either dissolves readily in the water used to wash it away or it evaporates [92, 93]. The varieties are divided into sweet and bitter cassavas depending on cyanogen content and genotypes. Moreover, cyanide is also found at low levels in certain seeds and stone fruits [18], such as apple (*Malus pumila*), mango (*Mangifera indica* L.), peach (*Prunus persica*), and bitter almonds (*Prunus dulcis*), so it is not specific to cassava tubers; however, intakes from cassava are generally much higher in the areas where it is commonly consumed.

Free linamarin from cassava may also be substantially absorbed into the blood and excreted intact in the urine without causing cyanide exposure [94–96], and this conclusion has been confirmed by others [97]. Less than a half of a linamarin dose is converted to cyanide. The released cyanide is partially biotransformed to thiocyanate, approximately one-quarter is excreted intact in the urine, and the metabolism of the remaining part is still unknown [97].

Linamarin and cyanide are common compounds in some other specific foods and from environmental exposures; the former is present in flax (*Linum usitatissimum*), butter bean (*Phaseolus lunatus*), white clover (*Trifolium repens*), and other plants [18], while the latter has been identified in the food of certain seeds and drupes, as well as in tobacco smoke [98]. The commonly consumed varieties of butter bean contain at least five times lower levels compared to cassavas [99].

Therefore, it seems that none of the compounds previously described is specific for cassava and cassava products intake. However, consumption of other sources of linamarin may be so limited that they would not interfere in areas with high cassava consumption. Flax containing cyanogenic glucosides may not be a common component of the diet, therefore intake may be limited compared to cassava; it is anticipated that linamarin may be low in species of flax used for consumption, although this is not yet documented. The linamarin level in butter bean is lower than in cassava, and white clover is not usually consumed or intakes are small, therefore linamarin detected in the urine can be proposed as promising biomarkers for cassava intake in many instances where exposure to the other sources can be ruled out or ignored as minimal.

Hernandez and co-workers [95] reported that the mean urinary linamarin was 0 before consumption, rapidly increased to 19 μmol/L as the maximum level, and then return to almost 0 at 12 h. The mean total urinary excretion was 28% and similar results have been reported by Carlsson et al. [97]. Due to the short half-life, no accumulation would be expected; however, this has not been investigated. Linamarin has been tested at several dose levels in a single subject with some evidence of dose-response; however, the evidence is too weak for a firm conclusion [97].

Thiocyanate is the predominant metabolite of cyanide by the sulfur-dependent enzyme, rhodanese (EC 2.8.1.1), when the subjects consume sufficient sulfur amino acids; otherwise, cyanide may conceivably be converted to cyanate, which may exacerbate the toxicity in populations deficient in sulfur-containing amino acids [86]. The urinary levels of thiocyanate can possibly be used to assess cyanide exposure. However, conversion into thiocyanate may be variable and a substantial fraction of cyanide may be converted into other metabolites in some individuals [100]. Besides, thiocyanate can be released by the breakdown of glucosinolates, which are produced by the enzyme, myrosinase (EC 3.2.1.147) in brassica vegetables, including broccoli, cabbage, cauliflower, turnip, and others. Lundquist and coworkers [101] identified another metabolite of cyanide, 2-amonithiazoline-4-carboxylic acid (ATC), which may be produced when the ingestion rate exceeds the conversion rate of cyanide to thiocyanate. Thus, the potential of thiocyanate as a BFI of exposure to cassava is questionable and needs further investigation, also considering environmental exposures. Cyanide itself may likewise not be a suitable biomarker to measure exposure to cassava, partially because it has a short half-life in plasma or whole blood and partially due to technical difficulties in sample preservation [102].

#### Jerusalem artichoke

The Jerusalem artichoke or topinambour (*Helianthus tuberosus*, L.) is a species of sunflower originating from North America and now grows widely across the temperate zone for its tuber, which is used as a root vegetable.

The tubers of Jerusalem artichoke contain about 80% water, 15% carbohydrate, and 1 to 2% protein; the tubers are also good sources of vitamins, especially vitamin B, vitamin C, and β-carotene, and have relatively high levels of folates or folic acid. The predominant storage carbohydrate is the fructan, inulin, accounting for approximately 50% of the dry weight [103]. In addition to inulin, the tubers contain some gentisic acid, heliangin, and spermine [9].

No specific compounds related to inulin degradation or to other compounds in Jerusalem artichoke are known to present to form putative BFIs or BFI precursors.

### Validation of candidate markers

A validation scoring scheme according to the BFI validation method [8] for candidate BFIs of tubers and tuber products is shown in Table 4. Only α-chaconine, α-solanine, solanidine, and total potato alkaloids as potential BFIs for potato, Pn 3-Caf·sop-5-glc and Cy 3-Caf·sop-5-glc for certain varieties for PSP, and linamarin for cassava were included since all other putative markers are still unlikely to be useful. The metabolites of AA and AC, diosgenin, cyanide and thiocyanate for potato, yam and cassava, respectively, were excluded based on this primary criterion.

**Table 4:** Validation scoring scheme for candidate tuber intake biomarkers

Food item	Metabolites	Biofluid locations	Questions^a^								
			1	2	3a	3b	4	5	6	7	8
Potato	Solanidine	Plasma	Y	U	U	U	U	U	Y	U	U
		Serum	Y	U	Y	Y	U	U	Y	Y	U
		Saliva	Y	U	U	U	U	U	Y	Y	U
	α-Solanine	Serum	Y	Y	Y	U	U	U	Y	Y	U
	α-Chaconine	Serum	Y	Y	Y	U	U	U	Y	Y	U
	Total potato alkaloids (α-solanine, α-chaconine and aglycone solanidine)	Serum	Y	U	U	U	U	U	Y	Y	U
		Saliva	Y	U	U	U	U	U	Y	Y	U
Sweet potato	Pn 3-Caf·sop-5-glc	Plasma	Y	U	U	U	U	U	Y	Y	U
		Urine	Y	N	Y	U	U	U	Y	Y	U
	Cy 3-Caf·sop-5-glc	Plasma	Y	U	U	U	U	U	Y	Y	U
		Urine	Y	U	Y	U	U	U	Y	Y	U
	Pn 3-Caf·sop-5-glc and Cy 3-Caf·sop-5-glc	Plasma	Y	U	Y	U	U	U	Y	Y	U
Cassava	Linamarin	Urine	Y	U	Y	U	U	U	Y	Y	Y

^a^The following nine validation criteria questions for biomarkers of food intake were used [8]: Q1: Is the marker compound plausible as a specific BFI for the food or food group (chemical/biological plausibility)? Q2: Is there a dose-response relationship at relevant intake levels of the targeted food (quantitative aspect)? Q3: Is the biomarker kinetics described adequately to make a wise choice of sample type, frequency, and time window (time-response)? a: The single-meal time-response relationship of the BFI has been described for a defined sample type and time window in a meal study. b: (The kinetics of the BFI after repeated intakes has been described for a defined sample type in a meal study) OR (accumulation of the BFI in certain sample types has been observed). Q4: Has the marker been shown to be robust after intake of complex meals reflecting dietary habits of the targeted population (robustness)? Q5: Has the marker been shown to compare well with other markers or questionnaire data for the same food/food group (reliability)? Q6: Is the marker chemically and biologically stable during biospecimen collection and storage, making measurements reliable and feasible? Q7: Are analytical variability (CV %), accuracy, sensitivity, and specificity known as adequate for at least one reported analytical method? Q8: Has the analysis been successfully reproduced in another laboratory (reproducibility)?

As already mentioned, the included compounds of Table 4, α-chaconine, α-solanine, solanidine, total potato alkaloids, are specific for Solanaceae plants, although it is still uncertain whether they may be present in other foods from the same family at levels of importance; Pn 3-Caf·sop-5-glc and Cy 3-Caf·sop-5-glc are known to be present in certain varieties of PSP and may not be generally applicable. Linamarin is not specific for cassava but cassava may be by far the most important source in areas where this tuber is commonly consumed.

Solanidine is the common aglycone of α-chaconine and α-solanine, as well as the major metabolite of absorbed potato alkaloids, and it has been identified in biofluids (plasma, serum, and saliva) by radioimmunoassay and HPLC; however, most aspects of biological validation are lacking, and the number of subjects may be seen as too low for thorough validation; for instance, one study recruited only two subjects to assess the cumulative aspect. α-Solanine, α-chaconine, and total potato alkaloids have been evaluated for their dose-response and time-response while other investigation to assess kinetics are scarce. Observational studies with these markers are needed to evaluate their robustness and reliability. Only a single study applied total potato alkaloids as a combined marker; in this study, serum and saliva were collected to monitor levels after intake of potato with normal as well as unusually high contents of alkaloids.

Regarding Pn 3-Caf·sop-5-glc and Cy 3-Caf·sop-5-glc, only two papers were found investigating their metabolism in humans following PSP intake; here, they were monitored both in plasma and urine by LC/MS or LC-ESI-MS/MS after PSP intake. No data are available for evaluation of most other aspects of validation and therefore further validation studies are needed.

For linamarin, several papers indicate that about half of the consumed amount is absorbed and excreted unmetabolized making the compound a potential BFI. However, since other food sources may be possible sources of linamarin, including butter beans, unrefined flax, and manioc, excretion of the compound may not be specific to cassava; anyway, linamarin may be a marker of sufficient specificity when the other food sources can be excluded.

Consequently, α-chaconine and/or α-solanine and/or solanidine seem to be promising markers for the assessment of potato intake, while Pn 3-Caf·sop-5-glc and/or Cy 3-Caf·sop-5-glc are candidate markers, only potentially specific for PSP. Finally, linamarin is a candidate marker of cassava intake when other foods, especially manioc and butter beans, can be excluded. Further validation studies, including analytical performance, kinetics, robustness, and reliability, are therefore needed to conclude on the usefulness of these biomarkers for potato, PSP, and cassava intake.

## Conclusions

In conclusion, probably the best candidate compounds to be considered as potentially specific BFIs for assessment of potato or potato products are glycoalkaloids, for cassava linamarin may be a candidate BFI, while for purple sweet potato consumption the best candidate BFIs are Pn 3-Caf·sop-5-glc and Cy 3-Caf·sop-5-glc. Much additional information and further study will be needed for their validation.

Solanidine has been reported to be the primary human metabolite in blood present from glycoalkaloids in potatoes, and studies exist on the short-term pharmacokinetics of glycoalkaloids and solanidine in humans; linamarin was found to be excreted in the urine in its unmetabolized form and may be considered as a candidate BFI when other food sources can be excluded; Pn 3-Caf·sop-5-glc and Cy 3-Caf·sop-5-glc are identified as metabolites of acylated anthocyanins in plasma and urine after PSP consumption. However, very little information is available from other types of human studies or from other foods containing acylated anthocyanins and further investigation on dose-response, kinetics and validation is needed.

Diosgenin seems to have some limited support as a putative BFI for assessment of yam intake, while no candidate markers were identified in the literature as specific for Jerusalem artichoke. However, little investigation has been done on human diosgenin metabolism and the compound may also have other significant food sources. These compounds are therefore still highly questionable as BFIs and further human studies and validations are needed.

For heated potato products, several mercapturic acids including unchanged AA, AAMA, GAMA, 3-HPMA, and CEMA were found as AA and AC metabolites but again these metabolites are not specific for intake of heated potato products since they are abundant also after intake of other heated foods. Other flavor compounds in heated potatoes, particularly the pyrazines, may exist with better specificity but they have not been investigated after exposure in humans, so their putative use as BFIs for fried potato products would need future research in the area. The use of markers of heated potato products in combination with glycoalkaloids may provide specificity to intake of potato fries and chips potentially facilitating intake estimation of different potato products in samples collected in epidemiological studies. This is an area of further research needed to help evaluate the relationship between exposures to tubers and tuber products and their relation to human health.

## Additional files

Additional file 1: **Figure S1.** Flow diagram of structured literature search for BFIs of sweet potato. **Figure S2.** Flow diagram of structured literature search for BFIs of yam. **Figure S3.** Flow diagram of structured literature search for BFIs of cassava. **Figure S4.** Flow diagram of structured literature search for BFIs of Jerusalem artichoke. (ZIP 1.70 mb)

Additional file 2: **Table S2.** List of studies reporting non-specific biomarker for tubers intake. (DOCX 16 kb)

Additional file 3: **Table S1.** Components reported in potatoes and heated potato products. (DOCX 19 kb)

## Abbreviations

AA: Unchanged acrylamide
AA-Hb: Hemoglobin adducts of acrylamide
AAMA: *N*-acetyl-S-(2-carbamoylethyl)-cysteine
AAMA-sul: *N*-acetyl-S-(2-carbamoylethyl)-l-cysteine-sulfoxide
ATC: 2-Aminothiazolin-4-carboxylic acid
BFIRev: Biomarker of Food Intake Reviews
BFIs: Biomarkers of food intake
CEMA: *N*-acetyl-*S*-(carboxyethyl)cysteine
Cy 3-Caf·sop-5-glc: Cyanidin 3-caffeoylsophoroside-5-glucoside
GA-Hb: Hemoglobin adducts of glycidamide
GAMA: *N*-acetyl-*S*-(2-hydroxy-2-carbamoylethylcysteine
HPMA: *N*-acetyl-*S*-(3-hydroxypropyl)cysteine
iso-GAMA: *N*-acetyl-*S*-(1-carbamoyl-2-hydroxyethyl)-l-cysteine
Pn 3-Caf·sop-5-glc: Peonidin 3-caffeoylsophoroside-5-glucoside
