# Supplementary figures and images for: Biomarkers of tuber intake
Source: Genes Nutr. 2019 Apr 2;14:9. doi: 10.1186/s12263-019-0631-0 (PMC6444566; doi:10.1186/s12263-019-0631-0)

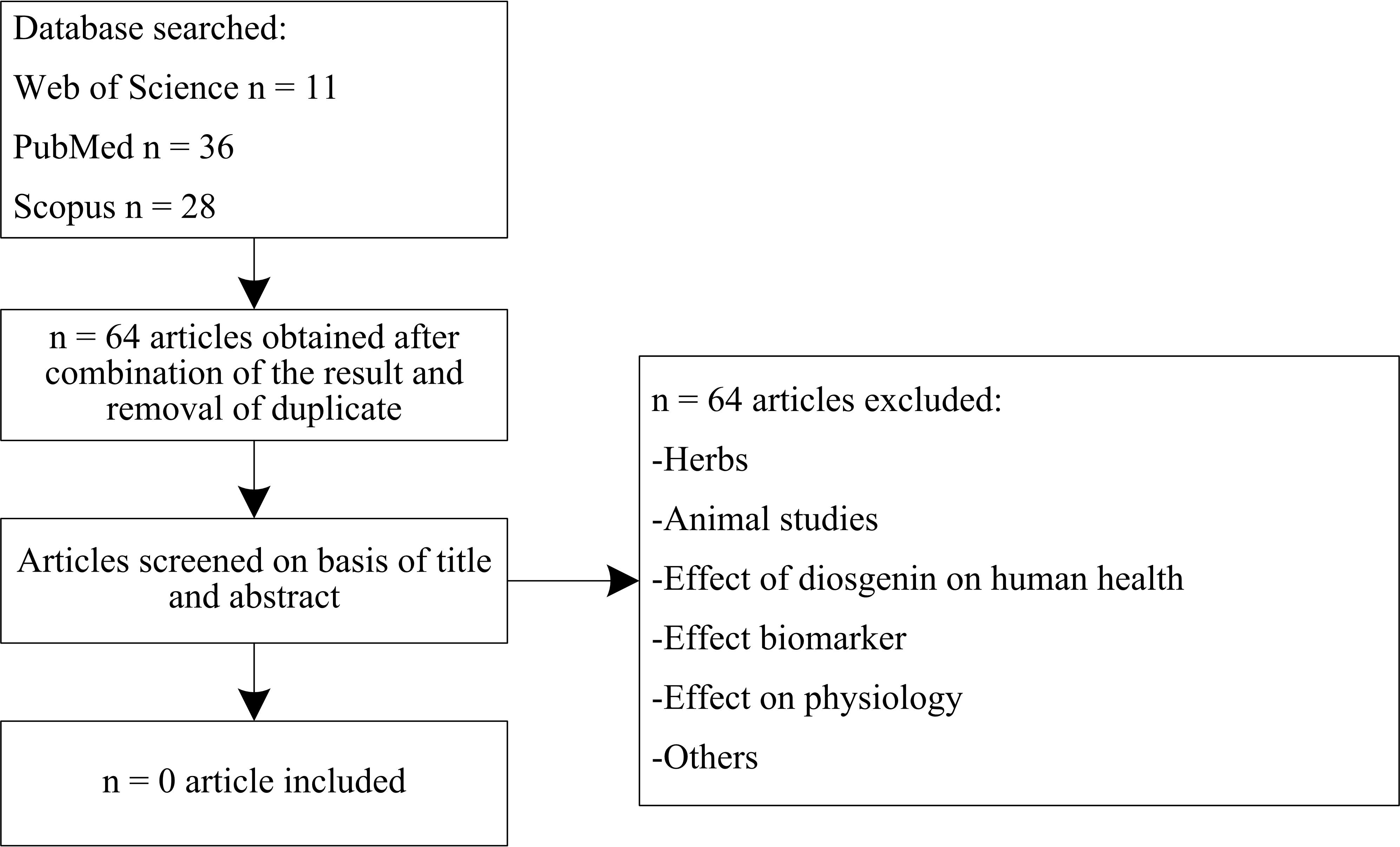

Supplement: Supplementary file 1 — Figure S1. Flow diagram of structured literature search for BFIs of sweet potato. Figure S2. Flow diagram of structured literature search for BFIs of yam. Figure S3. Flow diagram of structured literature search for BFIs of cassava. Figure S4. Flow diagram of structured literature search for BFIs of Jerusalem artichoke. (ZIP 1.70 mb) [file 12263_2019_631_MOESM1_ESM.zip › Fig S2.jpg]

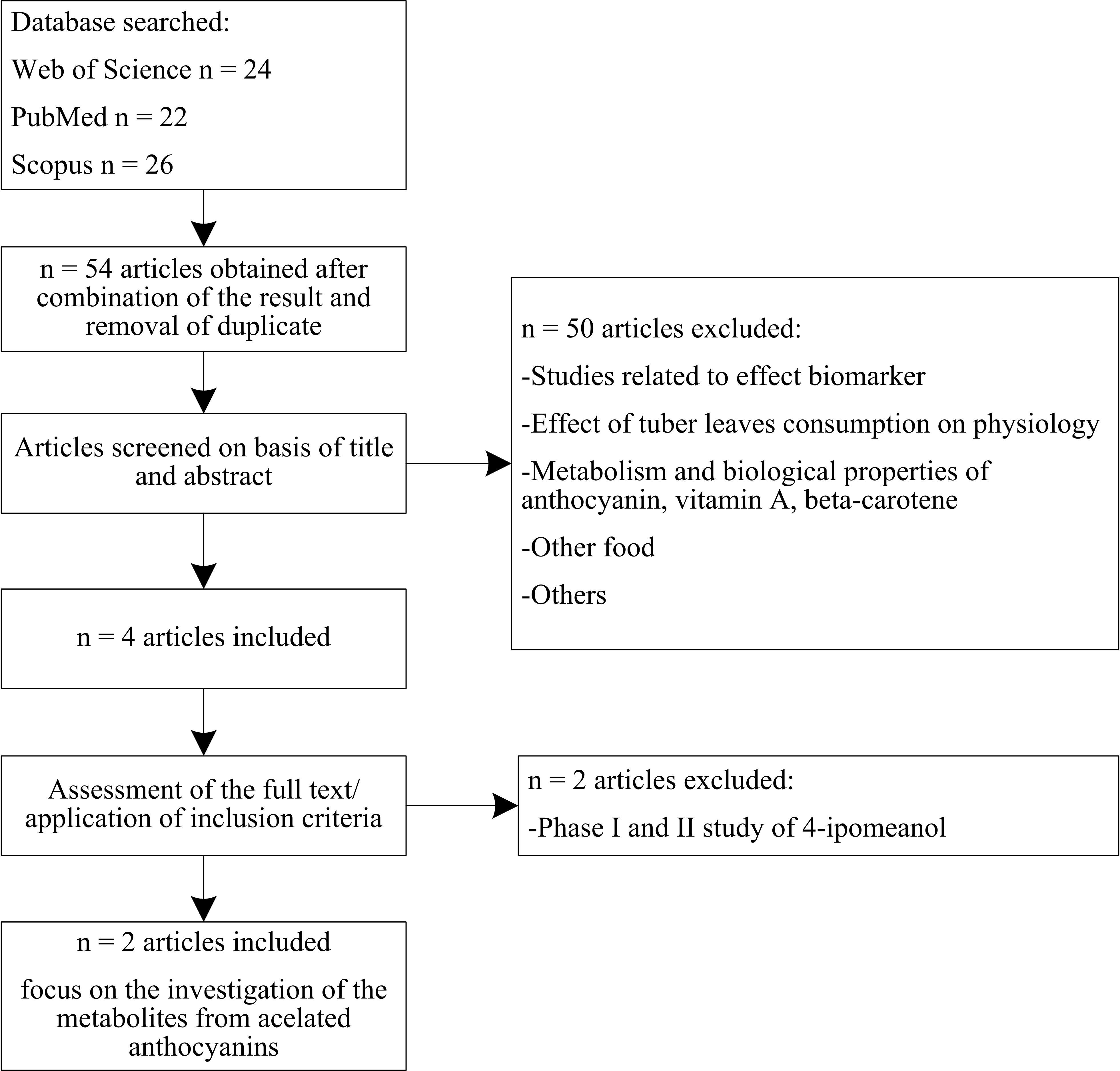

Supplement: Supplementary file 1 — Figure S1. Flow diagram of structured literature search for BFIs of sweet potato. Figure S2. Flow diagram of structured literature search for BFIs of yam. Figure S3. Flow diagram of structured literature search for BFIs of cassava. Figure S4. Flow diagram of structured literature search for BFIs of Jerusalem artichoke. (ZIP 1.70 mb) [file 12263_2019_631_MOESM1_ESM.zip › Figure S1.jpg]

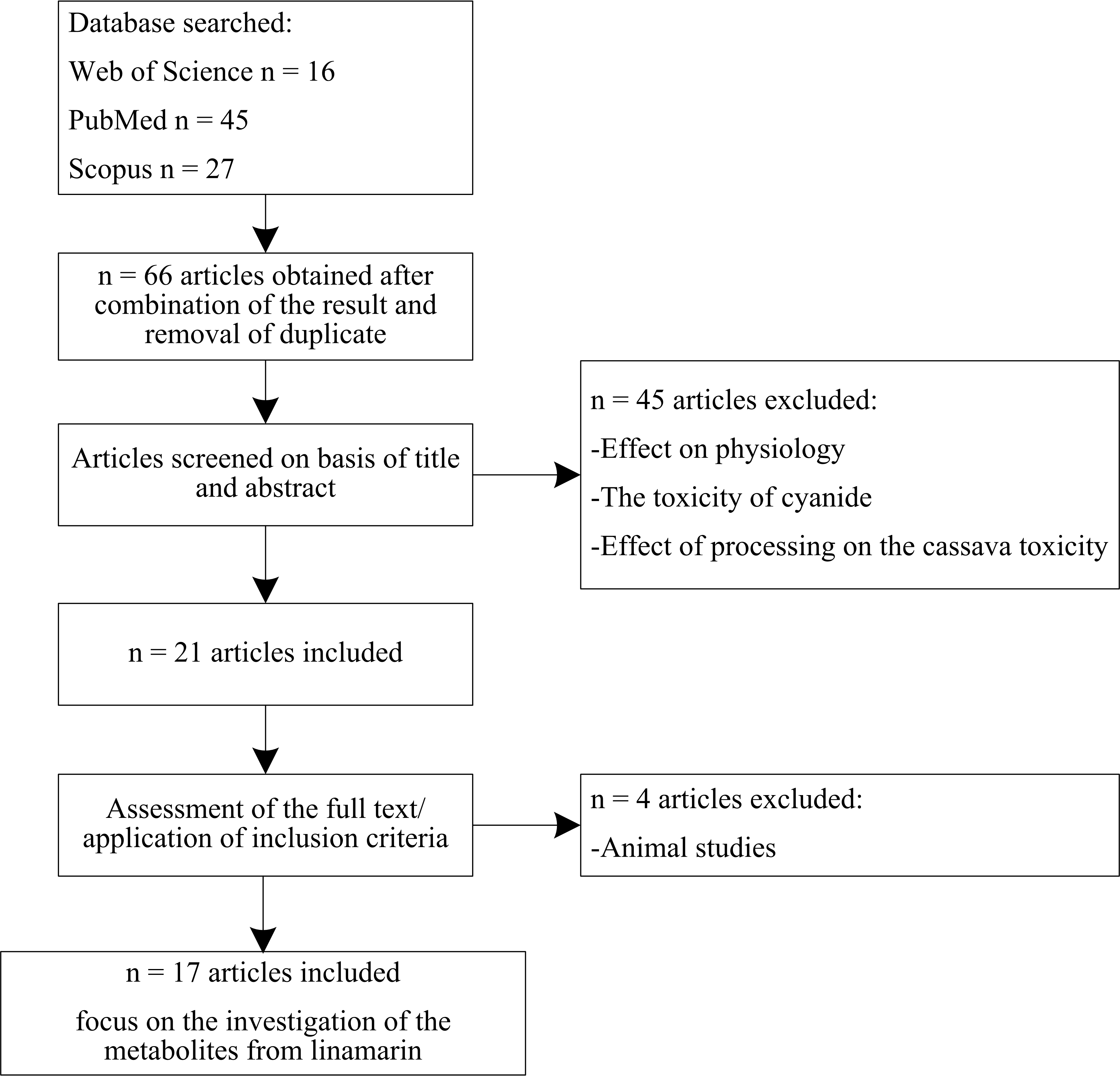

Supplement: Supplementary file 1 — Figure S1. Flow diagram of structured literature search for BFIs of sweet potato. Figure S2. Flow diagram of structured literature search for BFIs of yam. Figure S3. Flow diagram of structured literature search for BFIs of cassava. Figure S4. Flow diagram of structured literature search for BFIs of Jerusalem artichoke. (ZIP 1.70 mb) [file 12263_2019_631_MOESM1_ESM.zip › Figure S3.jpg]

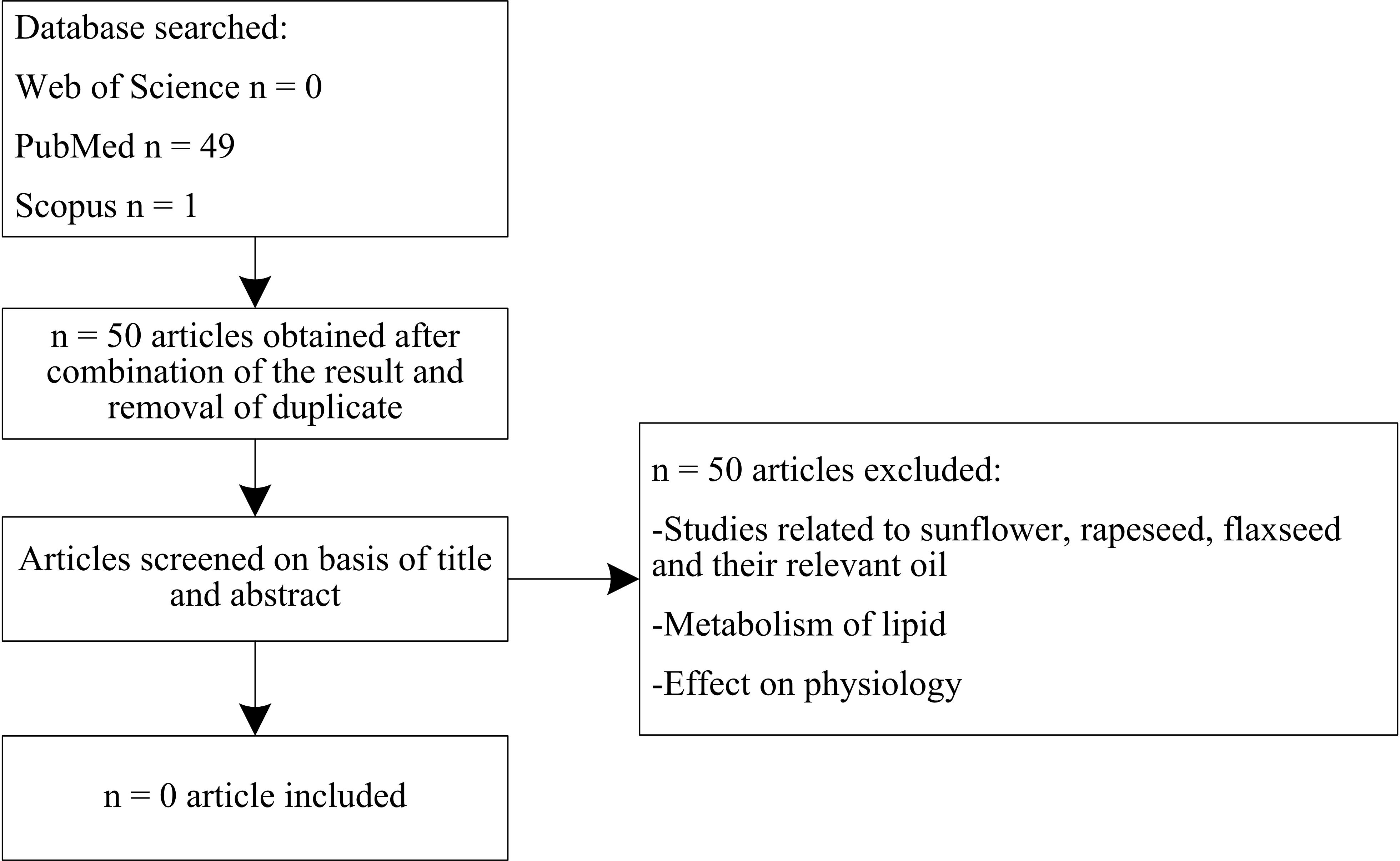

Supplement: Supplementary file 1 — Figure S1. Flow diagram of structured literature search for BFIs of sweet potato. Figure S2. Flow diagram of structured literature search for BFIs of yam. Figure S3. Flow diagram of structured literature search for BFIs of cassava. Figure S4. Flow diagram of structured literature search for BFIs of Jerusalem artichoke. (ZIP 1.70 mb) [file 12263_2019_631_MOESM1_ESM.zip › Figure S4.jpg]
