# Supplementary material for: Biomarkers of tuber intake
Source: Genes Nutr. 2019 Apr 2;14:9. doi: 10.1186/s12263-019-0631-0 (PMC6444566; doi:10.1186/s12263-019-0631-0)
Supplement: Supplementary file 2 — Table S2. List of studies reporting non-specific biomarker for tubers intake. (DOCX 16 kb) [file 12263_2019_631_MOESM2_ESM.docx]

**Table S2** List of studies reporting non-specific biomarker for tubers intake

| **Dietary factor** | **Study design** | **Sample type** | **Discriminating metabolites/Candidate biomarkers** | **Primary reference(s)** |
| --- | --- | --- | --- | --- |
| Potato chips | Human meal study | Blood (the beginning and the end of study) | AA-Hb;  GA-Hb | [1] |
| Potato chips | Crossover intervention study | Blood (prior and 24h postdose) | AA-Hb;  GA-Hb | [2] |
| Cassava consumption (Gari-based meals) | Observational study | Urine | Thiocyanate | [3] |
| Cassava consumption | Observational study | Urine | Thiocyanate | [4] |
| Cassava consumption | Observational study | Urine | Thiocyanate | [5] |
| Cassava consumption | Observational study | Urine;  serum | Thiocyanate | [6] |
| Cassava consumption | Observational study | Urine | Linamarin;  Thiocyanate | [7] |
| Cassava consumption (Stiff porridge) | Human meal study | Urine;  serum | Thiocyanate;  Linamarin | [8] |
| Cassava consumption (Stiff porridge made from short-soaked roots of bitter cassava varieties) | Observational study | Urine | Linamarin;  Thiocyanate | [9] |
| Cassava consumption | Observational study | Urine | ATC | [10] |
| Cassava consumption (Gari-based meals) | Human meal study | Serum;  urine | Thiocyanate | [11] |
| Cassava consumption | Observational study | Urine | Thiocyanate;  Linamarin | [12] |
| Cassava consumption (Gari-based meal) | Observational study | Serum | Thiocyanate | [13] |
| Cassava consumption (Gari-based meal) | Observational study | Whole blood; plasm (prior and 3-4 hours  postdose) | Cyanide | [14] |
| Cassava consumption (Cooked cassava) | Observational study | Urine;  plasma | Thiocyanate | [15] |
| Cassava consumption (Unsoaked cassava) | Intervention study | Serum;  urine | Thiocyanate | [16] |
| Cassava consumption (Cassava meals) | Human meal study | Plasma | Thiocyanate | [17] |
| Cassava consumption (especially cassava products like gari, fufu, and lafun) | Observational study | Urine;  serum | Thiocyanate | [18] |
| Cassava consumption (Boiled sweet cassava) | Human meal studies | Urine | Thiocyanate;  Linamarin | [19] |

Reference

1. Vesper HW, Licea-Perez H, Meyers T, Ospina M, Myers GL. Pilot Study on the Impact of Potato Chips Consumption on Biomarkers of Acrylamide Exposure. In: Chemistry and Safety of Acrylamide in Food. New York: Springer-Verlag; 2005. p. 89–96. doi:10.1007/0-387-24980-X_7.

2. Doroshyenko O, Fuhr U, Kunz D, Frank D, Kinzig M, Jetter A, et al. In vivo role of cytochrome P450 2E1 and glutathione-S-transferase activity for acrylamide toxicokinetics in humans. Cancer Epidemiol Biomarkers Prev. 2009;18:433–43.

3. Oluwole OSA., Oludiran AO. c. Normative concentrations of urine thiocyanate in cassava eating communities in Nigeria. Int J Food Sci Nutr. 2013;64:1036–41.

4. Banea JP, Nahimana G, Mandombi C, Bradbury JH, Denton IC, Kuwa N. Control of konzo in DRC using the wetting method on cassava flour. Food Chem Toxicol. 2012;50:1517–23.

5. Okafor PN. Assessment of cyanide overload in cassava consuming populations of Nigeria and the cyanide content of some cassava based foods. African J Biotechnol. 2004;3:358–61.

6. Okafor PN, Okorowkwo CO, Maduagwu EN. Occupational and dietary exposures of humans to cyanide poisoning from large-scale cassava processing and ingestion of cassava foods. Food Chem Toxicol. 2002;40:1001–5.

7. Chiwona-Karltun, T. Tylleskar, J. M L. Low dietary cyanogen exposure from frequent consumption of potentially toxic cassava in Malawi. Int J Food Sci Nutr. 2000;51:33–43.

8. Carlsson L, Mlingi N, Juma A, Ronquist G, Rosling H. Metabolic fates in humans of linamarin in cassava flour ingested as stiff porridge. Food Chem Toxicol. 1999;37:307–12.

9. Banea-Mayambu JP, Nkiabungu B, Tylleskar T, Rosling H. High cassava consumption without cyanide exposure in Kinshasa, in former Zaire. Ecol Food Nutr. 1998;37:363–77.

10. Lundquist P, Kagedal B, Nilsson L, Rosling H. Analysis of the Cyanide Metabolite 2-Aminothiazoline-4-Carboxylic Acid in Urine by High-Performance Liquid Chromatography. Anal Biochem. 1995;228:27–34.

11. Eminedoki DG, Monanu MO, Anosike EO. Thiocyanate levels of mainly dietary origin in serum and urine from a human population sample in Port Harcourt, Nigeria. Plant Foods Hum Nutr. 1994;46:277–85.

12. Brimer L, Rosling H. Microdiffusion method with solid state detection of cyanogenic glycosides from cassava in human urine. Food Chem Toxicol. 1993;31:599–603.

13. Oforofuo IAO, Omu AE. Serum thiocyanate levels in a normal population and in pregnancy in Benin City, Nigeria: preliminary report. Int J Gynecol Obstet. 1991;34:65–9.

14. Uwakwe AA, Monanu MO, Anosike EO. Whole blood cyanide levels of mainly dietary origin in a human population sample in Port Harcourt, Nigeria. Plant Foods Hum Nutr. 1991;41:117–24.

15. Pereira Bastos De Siqueira ME, Barros JM, Alves Oliveira A, Costa Esteves MT. Urinary and plasmatic reference levels for thiocyanates. Rev Farm Bioquim Univ Sao Paulo. 1996;32:21–7.

16. Ermans AM. Goitrogens of vegetable origin as possible aetiological factors in endemic goiter (author’s transl). Ann Endocrinol. 1981;42:435–8.

17. Akanji AO, Adeyefa I, Charles-Davies M, Osotimehin BO. Plasma glucose and thiocyanate responses to different mixed cassava meals in non-diabetic Nigerians. Eur J Clin Nutr. 1990;44:71–7.

18. Adewusi SRA, Akindahunsi AA. Cassava processing, consumption, and cyanide toxicity. J Toxicol Environ Health. 1994;43:13–23.

19. Hernández T, Lundquist P, Oliveira L, Cristià RP, Rodriguez E, Rosling H. Fate in humans of dietary intake of cyanogenic glycosides from roots of sweet cassava consumed in Cuba. Nat Toxins. 1995;3:114–7.
