# Supplementary material for: Biomarkers of tuber intake
Source: Genes Nutr. 2019 Apr 2;14:9. doi: 10.1186/s12263-019-0631-0 (PMC6444566; doi:10.1186/s12263-019-0631-0)
Supplement: Supplementary file 3 — Table S1. Components reported in potatoes and heated potato products. (DOCX 19 kb) [file 12263_2019_631_MOESM3_ESM.docx]

**Table S1** Components reported in potatoes and heated potato products

| **Category** | **Individual compounds** | | **References** |
| --- | --- | --- | --- |
| Phenolic acids | hydroxybenzoic acid derivatives | gallic acid | [1] |
|  |  | protocatechuic acid | [1] |
|  |  | vanillic acid | [1] |
|  |  | sinapic acid | [2] |
|  |  | syringic acid |  |
|  | hydroxycinnamic acid derivatives | chlorogenic acid  (5-O-caeoylquinic acid) | [1, 3] |
|  |  | cryptochlorogenic acid  (4-O-caeoylquinic acid) | [1] |
|  |  | neochlorogenic acid  (3-O-caeoylquinic acid) |  |
|  |  | caffeic acid | [1, 3] |
|  |  | p-coumaric acid | [1, 3] |
|  |  | ferulic acid | [1, 3] |
| Flavonoids | flavonols | quercetin | [4] |
|  |  | kaempferol | [5] |
|  |  | rutin | [6] |
|  | flavanols | catechin | [1, 5] |
|  |  | epicatechin | [5] |
|  | flavanones | erodictyol | [5] |
|  |  | naringenin | [5] |
|  | anthocyanins | | [6] |
| Glycoalkaloids | α-solanine | | [1, 7] |
|  | α-chaconin | | [1, 7] |
| Carotenoids | β-carotene | | [6] |
|  | lutein | | [6] |
|  | zeaxanthin | | [6] |
|  | violaxanthin | | [6] |
|  | neoxanthin | | [6] |
|  | β-cryptoxanthin | | [8] |
| Cooking process | pyrazine compounds | | [9, 10] |
|  | methional | | [11–13] |
|  | furans | | [14, 15] |
|  | acrylamide | | [14, 16–18] |
|  | acrolein | | [19] |
|  | short-chain fatty aldehydes | | [20] |
|  | strecker aldehydes | | [9] |
|  | thiazoles | | [21, 22] |
|  | oxazoles | | [21] |
|  | ethylcarbamate | | [23] |
|  | heterocyclic amines | | [23] |
|  | 5-hydroxymethylfurfural | | [23] |
|  | polycyclic aromatic hydrocarbons | | [23] |
|  | nitrosoamines | | [23] |

**Reference**

1. Mäder J, Rawel H, Kroh LW. Composition of phenolic compounds and glycoalkaloids α-solanine and α-chaconine during commercial potato processing. J Agric Food Chem. 2009;57:6292–7.

2. Mattila P, Hellström J. Phenolic acids in potatoes, vegetables, and some of their products. J Food Compos Anal. 2007;20:152–60.

3. Friedman M. Chemistry, biochemistry, and dietary role of potato polyphenols. A review. J Agric Food Chem. 1997;45:1523–40.

4. Mizuno M, Tsuchida H, Kozukue N, Mizuno S. Rapid Quantitative Free Quercetin Analysis and Distribution and Fruits of in Vegetables. Nippon Shokuhin Kogyo Gakkaishi. 1992;39:88–92.

5. Brown CR. Antioxidants in Potato. Amer J Potato Res. 2005;82:163–72.

6. Ezekiel R, Singh N, Sharma S, Kaur A. Beneficial phytochemicals in potato - a review. Food Res Int. 2013;50:487–96.

7. Friedman M, Roitman JN, Kozukue N. Glycoalkaloid and calystegine contents of eight potato cultivars. J Agric Food Chem. 2003;51:2964–73.

8. Breitbaupt DE, Bamedi A. Carotenoids and carotenoid esters in potatoes (Solanum tuberosum L.): New insights into an ancient vegetable. J Agric Food Chem. 2002;50:7175–81.

9. Martin FL, Ames JM. Formation of strecker aldehydes and pyrazines in a fried potato model system. J Agric Food Chem. 2001;49:3885–92.

10. Maga JA, Sizer CE. Pyrazines in foods. A Review. J Agric Food Chem. 1973;21:22–30.

11. Guadagni DG, Buttery RG, Turnbaugh JG. Odour thresholds and similarity ratings of some potato chip components. J Sci Food Agric. 1972;23:1435–44.

12. Ulrich D, Hoberg E, Neugebauer W, Tiemann H, Darsow U. Investigation of the boiled potato flavor by human sensory and instrumental methods. Am J Potato Res. 2000;77:111–7.

13. Petersen MA, Poll L, Larsen LM. Comparison of volatiles in raw and boiled potatoes using a mild extraction technique combined with GC odour profiling and GC-MS. Food Chem. 1998;61:461–6.

14. Mariotti M, Cortés P, Fromberg A, Bysted A, Pedreschi F, Granby K. Heat toxicant contaminant mitigation in potato chips. LWT - Food Sci Technol. 2015;60:860–6.

15. Mariotti MS, Granby K, Rozowski J, Pedreschi F. Furan: a critical heat induced dietary contaminant. Food Funct. 2013;4:1001–15.

16. Pedreschi F, Mariotti MS, Granby K. Current issues in dietary acrylamide: Formation, mitigation and risk assessment. Journal of the Science of Food and Agriculture. 2014;94:9–20.

17. Gökmen V, Palazoǧlu TK. Acrylamide formation in foods during thermal processing with a focus on frying. Food Bioprocess Technol. 2008;1:35–42.

18. Pedreschi F, Kaack K, Granby K. Reduction of acrylamide formation in potato slices during frying. LWT - Food Sci Technol. 2004;37:679–85.

19. Watzek N, Scherbl D, Feld J, Berger F, Doroshyenko O, Fuhr U, et al. Profiling of mercapturic acids of acrolein and acrylamide in human urine after consumption of potato crisps*. Mol Nutr Food Res. 2012.

20. Katsuta I, Shimizu M, Yamaguchi T, Nakajima Y. Emission of volatile aldehydes from DAG-rich and TAG-rich oils with different degrees of unsaturation during deep-frying. JAOCS, J Am Oil Chem Soc. 2008;85:513–9.

21. Coleman EC, Ho C-T, Chang SS. Isolation and identification of volatile compounds from baked potatoes. J Agric Food Chem. 1981;29:42–8.

22. Buttery RG, Ling LC. Alkylthiazoles in potato products. J Agric Food Chem. 1974;22:912–4.

23. Pedreschi F, Mariotti M S, Cortés P. Fried and Dehydrated Potato Products. In: Singh J, Kaur L, editors. Advances in Potato Chemistry and Technology. 2nd ed. Cambridge: Academic Press; 2016. p. 495.
